# A global database of lake surface temperatures collected by *in situ* and satellite methods from 1985–2009

**DOI:** 10.1038/sdata.2015.8

**Published:** 2015-03-17

**Authors:** Sapna Sharma, Derek K Gray, Jordan S Read, Catherine M O’Reilly, Philipp Schneider, Anam Qudrat, Corinna Gries, Samantha Stefanoff, Stephanie E Hampton, Simon Hook, John D Lenters, David M Livingstone, Peter B McIntyre, Rita Adrian, Mathew G Allan, Orlane Anneville, Lauri Arvola, Jay Austin, John Bailey, Jill S Baron, Justin Brookes, Yuwei Chen, Robert Daly, Martin Dokulil, Bo Dong, Kye Ewing, Elvira de Eyto, David Hamilton, Karl Havens, Shane Haydon, Harald Hetzenauer, Jocelyne Heneberry, Amy L Hetherington, Scott N Higgins, Eric Hixson, Lyubov R Izmest’eva, Benjamin M Jones, Külli Kangur, Peter Kasprzak, Olivier Köster, Benjamin M Kraemer, Michio Kumagai, Esko Kuusisto, George Leshkevich, Linda May, Sally MacIntyre, Dörthe Müller-Navarra, Mikhail Naumenko, Peeter Noges, Tiina Noges, Pius Niederhauser, Ryan P North, Andrew M Paterson, Pierre-Denis Plisnier, Anna Rigosi, Alon Rimmer, Michela Rogora, Lars Rudstam, James A Rusak, Nico Salmaso, Nihar R Samal, Daniel E Schindler, Geoffrey Schladow, Silke R Schmidt, Tracey Schultz, Eugene A Silow, Dietmar Straile, Katrin Teubner, Piet Verburg, Ari Voutilainen, Andrew Watkinson, Gesa A Weyhenmeyer, Craig E Williamson, Kara H Woo

**Affiliations:** 1 Department of Biology, York University, Toronto, Ontario, Canada M3J 1P3; 2 California University of Pennsylvania, California, Pennsylvania 15419, USA; 3 U.S. Geological Survey, Center for Integrated Data Analytics, Middleton, Wisconsin 53562, USA; 4 Department of Geography-Geology, Illinois State University, Normal, Illinois 61790, USA; 5 NILU—Norwegian Institute for Air Research, Kjeller 2027, Norway; 6 Center for Limnology, University of Wisconsin-Madison, Madison, Wisconsin 53706, USA; 7 Center for Environmental Research, Education and Outreach, Washington State University, Pullman, Washington 99164, USA; 8 NASA Jet Propulsion Laboratory, California Institute of Technology, California 91109, USA; 9 LimnoTech, Ann Arbor, Michigan 48108, USA; 10 Department of Water Resources and Drinking Water, Eawag (Swiss Federal Institute of Aquatic Science and Technology), Duebendorf CH-8600, Switzerland; 11 Leibniz-Institute of Freshwater Ecology and Inland Fisheries, Berlin D-12587, Germany; 12 Environmental Research Institute, University of Waikato 3240, New Zealand; 13 French National Institute for Agricultural Research (INRA), Station d’hydrobiologie lacustre (UMR CARRTEL), Thonon les Bains 74200, France; 14 University of Helsinki, Lammi Biological Station, Helsinki FI-16900, Finland; 15 Large Lakes Observatory, University of Minnesota Duluth, Duluth 55812, Minnesota, USA; 16 Vale Living with Lakes Centre, Ontario Ministry of the Environment and Climate Change, Sudbury, Ontario, Canada P3E 2C6; 17 U.S. Geological Survey Fort Collins Science Center, Colorado State University, Fort Collins, Colorado 80523, USA; 18 Water Research Centre, School of Biological Sciences, The University of Adelaide, Adelaide 5005, Australia; 19 Nanjing Institute of Geography and Limnology, Chinese Academy of Sciences 210008, China; 20 Australian Water Quality Center, SA Water Corporation 5001, Australia; 21 Research Institute for Limnology, University Innsbruck, Mondsee A-5310, Austria; 22 Department of Atmospheric and Environmental Sciences, University at Albany, State University of New York, Albany 12222, New York, USA; 23 Archbold Biological Station, Venus, Florida 33960, USA; 24 Fisheries Ecosystems Advisory Services, Marine Institute, Newport, Co. Mayo Ireland; 25 Environmental Research Institute, University of Waikato, Hamilton 3240, New Zealand; 26 Florida Sea Grant and University of Florida Institute of Food and Agricultural Sciences, Gainesville, Florida 32611, USA; 27 Melbourne Water Corporation, Victoria 3001, Australia; 28 LUBW Landesanstalt für Umwelt, Messungen und Naturschutz Baden-Württemberg, Institut für Seenforschung, Langenargen D-88045, Germany; 29 Department of Natural Resources, Cornell University, Ithaca, New York 14853, USA; 30 International Institute of Sustainable Development—Experimental Lakes Area, Winnipeg, Manitoba, Canada R3B 2L6; 31 Central Nebraska Public Power and Irrigation District, Holdredge, Nebraska 68949, USA; 32 Scientific Research Institute of Biology, Irkutsk State University, Irkutsk 664003, Russia; 33 U.S. Geological Survey Alaska Science Center, Anchorage, Alaska 99508, USA; 34 Institute of Agricultural and Environmental Sciences, Estonian University of Life Sciences Rannu, Estonia 61117; 35 Department of Experimental Limnology, Leibniz Institute of Freshwater Ecology & Inland Fisheries, Berlin 12587, Germany; 36 Wasserversorgung der Stadt Zurich (WVZ), Zurich CH-8021, Switzerland; 37 Lake Biwa Environmental Research Institute, Otsu, Shiga 520-0022, Japan; 38 Finnish Environment Institute, Helsinki FI-00250, Finland; 39 NOAA/Great Lakes Environmental Research Laboratory, Ann Arbor, Michigan 48108, USA; 40 Centre for Ecology and Hydrology, Bush Estate, Midlothian, Scotland EH260QB, UK; 41 Department of Ecology, Evolution and Marine Biology, University of California, Santa Barbara, California 93106, USA; 42 Department of Biology, University of Hamburg, Hamburg 22609, Germany; 43 Hydrology Laboratory, Limnology Institute, Russian Academy of Sciences, St Petersburg, Russian Federation 196105; 44 Centre for Limnology, Institute of Agricultural and Environmental Sciences, Estonian University of Life Sciences, Tartumaa 61117, Estonia; 45 Amt fur Abfall, Wasser, Energie und Luft, Kanton Zurich, Zurich CH-8005, Switzerland; 46 Institute of Coastal Research, Helmholtz-Zentrum Geesthacht, Geesthacht 21502, Germany; 47 Dorset Environmental Science Centre, Ontario Ministry of the Environment and Climate Change, Dorset, Ontario, Canada P0A 1E0; 48 Department of Earth Sciences, Royal Museum for Central Africa, Tervuren 3080, Belgium; 49 Israel Oceanographic and Limnological Research, The Lake Kinneret Limnological Laboratory, Migdal 14950, Israel; 50 National Research Council, Institute of Ecosystem Study, Verbania, Pallanza 28922, Italy; 51 IASMA Research and Innovation Centre, Istituto Agrario di S. Michele all'Adige, Fondazione E. Mach, S. Michele all’Adige (Trento) 38010, Italy; 52 Institute for Sustainable Cities, City University of New York, New York 10065, USA; 53 School of Aquatic and Fishery Sciences, University of Washington, Seattle, Washington 98195, USA; 54 UC Davis Tahoe Environmental Research Center, Incline Village, Nevada 95616, USA; 55 Sydney Catchment Authority, Penrith, NSW 2750, Australia; 56 Department of Biology, Universitat Konstanz, Konstanz 78464, Germany; 57 Department of Limnology and Biological Oceanography, University of Vienna, Vienna A-1090, Austria; 58 National Institute of Water and Atmospheric Research, Hamilton 1010, New Zealand; 59 Department of Biology, University of Eastern Finland, Kuopio 70211, Finland; 60 Seqwater, Ipswich QLD 4305, Australia; 61 Department of Ecology and Genetics/Limnology, Uppsala University, Uppsala 75236, Sweden; 62 Department of Biology, Miami University, Oxford, Ohio 45056, USA

**Keywords:** Climate-change ecology, Limnology, Climate change

## Abstract

Global environmental change has influenced lake surface temperatures, a key driver of ecosystem structure and function. Recent studies have suggested significant warming of water temperatures in individual lakes across many different regions around the world. However, the spatial and temporal coherence associated with the magnitude of these trends remains unclear. Thus, a global data set of water temperature is required to understand and synthesize global, long-term trends in surface water temperatures of inland bodies of water. We assembled a database of summer lake surface temperatures for 291 lakes collected *in situ* and/or by satellites for the period 1985–2009. In addition, corresponding climatic drivers (air temperatures, solar radiation, and cloud cover) and geomorphometric characteristics (latitude, longitude, elevation, lake surface area, maximum depth, mean depth, and volume) that influence lake surface temperatures were compiled for each lake. This unique dataset offers an invaluable baseline perspective on global-scale lake thermal conditions as environmental change continues.

## Background & Summary

Freshwater ecosystems are vulnerable to the effects of global environmental change^1^. Lakes are sentinels of climate change^2^ and are an effective indicator of a limnological response to global climate change, as they integrate climatic and landscape factors^3^. Lake surface temperatures can directly or indirectly influence physical, chemical, and biological processes in a lake, including the temperature of the photosynthetic zone^4^, water-column stability, lake primary productivity^5^, fish species range shifts^3^, and species interactions^6^.

Climatic factors, including air temperature, cloud cover, and solar radiation, in addition to geomorphometric factors, such as lake surface area and depth, influence surface water temperatures in lakes^7,8^. Here, we present a database of 25-year (1985–2009) lake surface water temperatures from 291 lakes around the world, collected by either *in situ*, satellites, or both, and some of the main corresponding climatic and geomorphometric factors that are relevant to lake surface water temperatures.

Between 1986 and 2005, global air temperatures have increased by 0.61 °C (ref. 9). Water temperatures have increased over recent decades coinciding with changing air temperatures, with a few exceptions. Generally, water temperatures tend to coincide with regional air temperatures, warming or cooling at similar rates to that of air temperatures^10,11^. However, there are some regions, such as around the Great Lakes region of North America and northern Europe, in which bodies of water are warming more rapidly than ambient air^11,12^.

Solar radiation is a key component of lake heat budgets^13^. Increases in the amount of incoming solar radiation increase average lake temperatures^14^. Cloud cover can reduce incident solar radiation, but also result in greater longwave (atmospheric) radiation^15^. Thus, the influence of cloud cover on lake temperatures can be bidirectional and complex.

Lake-specific properties, including depth, surface area, and volume can mediate the effects of these climate drivers and influence lake temperatures^13^. For example, surface water temperatures have been found to be inversely correlated with mean depth^8,16^. In general, shallower lakes tend to warm more rapidly with higher surface water temperatures compared to deep lakes that have greater heat storage capacities^8^. However, at broad spatial scales, climatic factors have a larger influence on surface water temperatures than lake morphology^17^.

The motivation to assemble a global database of lake surface water temperatures, climatic forcing variables, and geomorphometric characteristics was to address two key scientific issues by the Global Lake Temperature Collaboration (www.laketemperature.org): i) What are the global and regional patterns of water temperature changes over the past 25 years and are they coherent over spatial and temporal scales?; and ii) Which climatic forcing variables and geomorphometric factors are most important in driving changes in water temperature, i.e., do similar lake types exhibit similar rates of warming or cooling globally? This novel dataset can be used to address additional research questions including those pertaining to understanding the mechanistic drivers of surface water temperature warming and ecological consequences of changes in lake temperatures.

## Methods

### *In situ* water temperatures

#### *In situ* data

**Figure 1:**
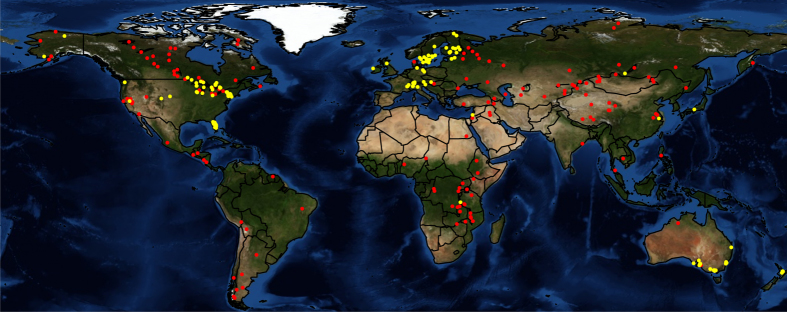
Map of the lakes included in the GLTC dataset. Yellow—*in situ* sampled lakes; Red—satellite sampled lakes.

**Figure 2:**
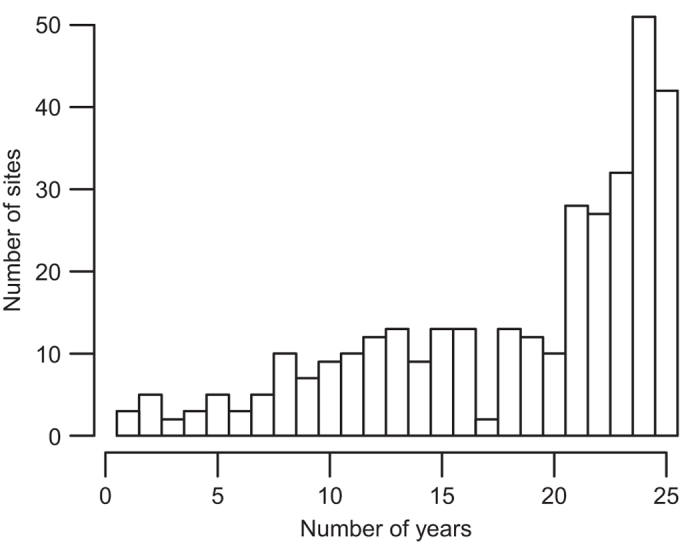
Histogram of temporal data coverage of summer mean water temperatures for sites within the GLTC dataset.

We compiled *in situ* surface water temperatures and corresponding lake geomorphology and climatic data from 151 lakes (Figure 1; Tables 1 and 2; Data Citation 1). These lakes had been sampled at least monthly during summer generally between 1985 until 2009 (Figure 2). Surface water was defined as between 0 and 1 m below the surface for most lakes. Below we describe the sampling methodology for each lake or group of lakes contributed by each data provider, organized by geographic region.

### Western North America

Lake Washington (King County, Washington) and Lake Aleknagik (Dillingham Census Area, Alaska) are both located in the United States. The lakes were sampled weekly to monthly, depending on the year, at a depth range of 0–60 m using a combination of continuous sampling and discrete profile measurement techniques. While samples were obtained from a single station near the centre of Lake Washington, they were obtained from six stations distributed across Lake Aleknagik. Water temperatures were recorded between 10:00 and 18:00 with a Kahl thermistor, although a Yellow Springs Instrument Co. (YSI) sonde 600XLM V2 was used starting in 1999 in Lake Aleknagik and YSI 660V2–4 in Lake Washington^18,19^.

Toolik Lake, a multibasin kettle lake in the Alaskan Arctic in the northern foothills of the Brooks Range, United States, is a major study site of the Arctic Long Term Ecological Research program (http://ecosystems.mbl.edu/ARC/dataprotocol/Arctic LTERIM.html). Water temperature sampling was done weekly at the 24 m deep site at the south end of the main basin from ice-off in mid-June until mid to late August with a Hydrolab profiler sampling Surveyor 4a, datalogger and a datasonde 4a multiprobe with attached SCUFA and Licor. Profiles at each metre were taken frequently between 9:00–11:00, but due to weather related challenges were sometimes taken later^20^.

Lake Tahoe is located in Sierra Nevada, United States. It was sampled weekly or monthly at 13 depths down to 450 m until 2005 and continuously since then. Specifically, temperatures were measured at depths of 0, 2, 5, 10, 15, 20, 30, 50, and 100 m at the centre of the lake^21^. Water temperatures were recorded at 10:00 with reversing thermometers until 2005 and with Seabird SBE25 since 2005 (ref. 22).

The Loch, located in Loch Vale watershed northwest of Denver, Colorado, United States, was sampled at the outlet directly upstream of Parshall Flume (Loch Outlet). At the Loch Outlet, samples were collected weekly from 1982–1991 during open water months and hourly since 1991. Water temperature samples were collected at 10 cm depth in the late morning to early afternoon. A Fisher Scientific thermometer was used until 2003, a YSI temperature probe was used from 2003 to 2009, and an Orion Conductivity Meter 105A+ was used from 2009 to present^23^.

Sky Pond, located in Loch Vale watershed northwest of Denver, Colorado, United States, was sampled at Sky Pond outlet where Sky Pond drains to Icy Brook. Samples were collected weekly from 1982–1991 during open water months and monthly during open water months since 1991. Water temperature samples were collected at 10 cm depth in the early afternoon. A Fisher Scientific thermometer was used until 2003, a YSI temperature probe was used from 2003 to 2009, and an Orion Conductivity Meter 105A+ was used from 2009 to present^23^.

Lake McConaughy is a regulated reservoir located near Ogallala, Nebraska, United States. It was sampled at a variable frequency over the study period, from once per week to once per two months, at 1 m increments from the surface to the bottom using a YSI 56. The samples were obtained from near the Kingsley Dam^24^.

### Northeastern North America

Water temperatures in Lakes Superior, Huron, Michigan, and Erie in North America were recorded every 10 min to hourly at a depth of 0.6–1 m. The samples were obtained from three different sites in Lake Superior^12^, two sites each on Lakes Huron and Michigan, and one site on Lakes Erie. Water temperatures were recorded using 2–3 m surface buoys^25^, usually ARES or AMPS payload (http://www.ndbc.noaa.gov/rsa.shtml) and various hulls.

Trout Lake, Crystal Lake, Sparkling Lake, Allequash Lake, Big Muskellunge Lake are in northern Wisconsin, United States. Lake Mendota, Lake Monona, Lake Wingra, and Fish Lake are in southern Wisconsin. These lakes were sampled fortnightly, typically at 1 m intervals including the surface by the North Temperate Lakes Long Term Ecological Research program^26^. Temperatures were obtained from a consistent sampling location at or near the deepest portion of the lake. Sampling was performed during the daytime, typically between 9:00 and 15:00 with a YSI Model 58 tempDO metre (https://lter.limnology.wisc.edu/about/lakes).

Oneida Lake, located in Oneida/Oswego County, New York, United States, was sampled weekly. Surface temperature was measured at eight standard locations throughout Oneida Lake, although the Shackelton Point site approximately 0.7 km from the south shore of the lake consisted of the longest time series. Water temperatures were recorded between 8:00 and 12:00 using a YSI profiler from 1975–1994, a Hydrolab Datasonde II profiler from 1995–2010, and a Hydrolab Datasonde 5 profiler in 2011. Complete water temperature profiles are available https://knb.ecoinformatics.org/#view/kgordon.35.49.

Water temperatures from Cannonsville, Pepacton, Rondout, Neversink, Schoharie, Ashokan East, Ashokan West, and Kensico, in New York, United States were generally sampled monthly (although weekly in some years). Water temperatures were measured from within 1 m from the lake water surface in each reservoir^27^. Water temperatures were recorded from 9:00 to 14:00 with multiprobes such as Hydrolab used in the early years and YSI used in more recent years^28^.

Lake Giles and Lake Lacawac are in Pennsylvania, United States, and were sampled weekly to monthly at a depth of 0.5 m. Both lakes were sampled from the surface (0 m) to the bottom (20–23 m for Lake Giles and 10–13 m for Lake Lacawac). The samples were obtained from a designated mid-lake sampling location using a YSI temperature/oxygen meter (Model 58), Biospherical Cosine Irradiance (BIC) radiometer, or a Profiling Ultraviolet (PUV) radiometer^29^.

Six lakes of the Experimental Lakes Area (L223, L224, L227, L239, L240, and L302s), located in Northwestern Ontario, Canada, were sampled daily from 1979-present at 1 m depths^30^. Bi-weekly depth profiles were generated every meter, except immediately above the thermocline and in the metalimnion where 0.25 m depth increments were used. The samples were obtained from the region with greatest water depth within each lake. Water temperature profiles were obtained during the daylight hours between 9:00 to 14:00 using a Peabody Ryan Model J Thermograph between 1980–88, a Richard Brancher Temperature Logger XL-800 between 1989–97, a Hobo onset stow away logger between 1998–2007, and a Hobo water temperature pro V2 from 2008 to present. Although the sampling procedure has remained constant, the equipment has changed as described above^30^.

Blue Chalk, Chub, Crosson, Dickie, Harp, Heney, Red Chalk East, Red Chalk Main, and Plastic Lakes are located near the Dorset Environmental Science Centre in Ontario, Canada. All lakes were sampled fortnightly in 1985. From 1986 to 1991, Crosson, Harp, Heney and Plastic Lakes were sampled fortnightly throughout the ice-free season, with the remaining lakes sampled monthly. From 1991 to the 2009, all lakes were sampled monthly through the ice-free season, with the exception of Dickie, Harp and Plastic Lakes which were sampled every two weeks. Generally, the lakes were sampled from the surface to the maximum depth at 1 m intervals at the deepest sampling location between 10:00 to 14:00, using analog and digital versions of the YSI temperature/DO probe (YSI models 58 and 95) (ref. 31).

Clearwater, Hannah, Lohi, Middle, Sans Chambre, Swan and Whitepine Lakes are all located near Sudbury, Ontario, Canada. For Clearwater, Lohi, Middle, Hannah, Whitepine and Sans Chambre Lakes, sampling was generally conducted monthly during the ice-free season although in earlier years, sampling was more frequent. For Swan Lake, sampling was generally conducted every two weeks during the ice-free season. The lakes were sampled at every meter from the surface to the bottom. Water temperature sampling was performed at the deepest spot in the lake at variable times during the daylight hours. YSI brand dissolved oxygen and temperature meters have been used to record water temperatures^32^.

### Southeastern North America

Lakes Apopka (Orange and Lake Counties), Beauclair (Lake County), Blue Cypress (Indian River County), Carlton (Lake County), Crescent Lake (Putnam and Flagler Counties), Denham (Lake County), Dora (Mount Dora), Eustis (Lake County), George (Lake County), Griffin (Lake County), Harney (between Volusia and Seminole Counties), Harris (Lake County), Jesup (Seminole County), Lochloosa (Alachua County), Newnans (Alachua County), Okeechobee (southeastern Florida), Orange (Alachua County), Poinsett (Brevard County), and Washington (Washington, Florida) (Brevard County) are located in Florida, United States and from the South Florida and St John’s River Water Management Districts. These lakes were sampled monthly at an approximate depth of 0 m from highly mixed water columns at the middle of the lake. After 1992, sampling depth for Lake Ocheechobee changed from 0 to 0.5 m. Water temperature sampling was performed in the morning with a YSI multi-parameter *in situ* water analyzer. Water temperatures were measured *in situ* with either a YSI or Hydrolab data sonde in the morning, and calibration was done on the day or week of sampling against National Bureau of Standards thermometers.

The Lake Annie Buoy is located near the centre, over the deepest part (20.4 m) of Lake Annie, Highlands County, Florida. Sampling was carried out with automated thermistors, located at 1 m intervals from the top to the bottom of the lake. An Ecolab Templine was used between February 14, 2008 and September 15, 2010 and a Fondriest thermistor chain was used from February 14, 2011 to present. The same procedure was used for cleaning and calibration of both instruments. Data are publicly available online at http://www.archbold-station.org.

### Europe

Lough Feeagh, located in County Mayo, Ireland was sampled daily from the surface at less than 1 m depth. Water temperatures were recorded at a site 60 m along the outflow draining straight out of the lake^33^. Water temperature sampling was conducted continuously (*in situ* paper or sensor record), but compiled daily at midnight. From 1960–2004, a paper chart recorder with a mercury thermometer was used. From 2004 to present, a StowAway TidbiT temperature data logger from Onset (TBI32-05+37) was used.

Loch Leven, situated in Perth and Kinross, Scotland, United Kingdom, was sampled biweekly, from the subsurface at an approximate depth of 0.01 m. The samples were obtained from the Reed Bower sampling site which is an open water site that has a water depth similar to the average depth of the lake and remains accessible in most weather conditions. Water temperatures were recorded in the morning, typically between 10:00–12:00 with a mercury-in-glass thermometer in a Ruttner closing bottle^34^.

Lake Annecy and Lake Bourget (located in France) and Lake Geneva located in France and Switzerland were sampled one or two times per month from the middle of the lake at the deepest point. Lake Annecy was sampled at depths of 2.5 and 5 m, Lake Bourget at depths of 0, 2, and 5 m, and Lake Geneva at 0, 2.5, and 5 m. Water temperatures were recorded in the morning between 9:00 and 11:30 using thermometers and multiparameter probes. In Lake Annecy, the Standart-ECO-Probe Version II was used from 1991 to 2002 and in 2006. From 2003 to 2005 and in 2007, the CTD 90 multiparameter probe was used. In 2008 and 2009, the CTD 90 M multiparameter probe was used. In Lake Bourget, water temperature was measured with the ISMA probe DNTC (Ponselle) in 1985. From 1986 to 1998 the multiparameter probe Meerestechnik Elektronik (ECO 236) was used. From 1999 to 2002 the CTD SBE 19 SeaCAT Profiler was used and thereafter the CTD SBE 19plus V2 SeaCAT profiler. In Lake Geneva, the water temperature was measured with a thermometer until 1991; starting from 1992 multiparameter probes were used. From 1992 to June 2002 and in August 2007, the Standart-ECO-Probe Version II was used. The CTD 90 multiparameter probe was used from July 2002 to February 2006, from July 2006 to July 2007, from September 2007 to June 2008, from December 2008 to March 2009 and from September 2009 to December 2009.The CTD 90 M multiparameter probe was used from August 2008 to October 2008 and from April 2009 to August 2009 (ref. 35).

Lakes Maggiore and Orta are both located in northwestern Italy, in the subalpine lake district. The lakes were sampled monthly at the deepest points. Twelve (from 0 to 360 m) and nine (from 0 to 140 m) depths were considered for Lake Maggiore and Orta, respectively. Water temperatures were recorded between 10:00 and 12:00 depending on the sampling day using reversing thermometers connected to the bottle for water sampling^36^.

Lake Garda, located in northern Italy was sampled monthly from the surface at a depth of 0.5 m. The samples were obtained from the centre of the lake, in the zone of maximum depth. Water temperatures were measured between 11:30 and 14:30 depending on the sampling day and meteorological conditions. Various multiparameter probes were used and constantly controlled by the manufactures (Idronaut in Italy and Seabird in United States), giving coincident results. Specifically, from 1991 to 1997, the Idronaut Ocean Seven 401 was used, from 1998 to 2008, the Seacat SBE 19-03 was used, while from 2009-present, the Idronaut Ocean Seven 316Plus^37^ was used.

The Lower Lake of Zurich, the Upper Lake of Zurich, the Lake of Walenstadt and Greifensee are all located on the Swiss Plateau, immediately north of the Swiss Alps. Water temperatures were measured about 0.3 m below the surface, usually at the deepest point of each lake^38^. Sampling intervals were approximately monthly with the exception of the Upper Lake of Zurich from 2006 (3-monthly) and the Lake of Walenstadt from 2001 (3-monthly). Water temperatures in the Lower Lake of Zurich, Upper Lake of Zurich and Lake of Walenstadt were measured from January 1985 to January 2001 using three different negative temperature coefficient (NTC) thermistors; from February 2001—July 2008 using an FLP-10 multisonde (DMP AG, Switzerland); and from August 2008 onwards using a Hydrolab DS5 multisonde (OTT Hydromet GmbH, Germany). On rare occasions from 2001 onwards when the multisondes were unavailable, the NTC thermistors were again employed. Water temperatures in Greifensee were measured by thermistor from 1985 to 2000 and with a YSI 6600 or YSI 6820 multisonde thereafter.

Lake Plusssee, located in northern Germany, was sampled weekly since 1985 at depths of 0 to 15 m with intervals of 1 m and at 20, 25, and 26 m. The samples were obtained from the middle of the lake, above the deepest spot. Water temperature sampling was usually performed in the mornings with a temperature probe (OXI191 from WTW)^39^.

Lakes Mondsee, Neusiedler, and Wörther (Woerther See) are located in Austria and sampled daily at a depth of 0.2 m. The samples were obtained from the lake level gauging station. Water temperature sampling was performed between 8:00 to 10:00, usually at 9:15. In the earlier years, a thermometer assembled in a Ruttner water sampler was used, and following 2000, temperature was recorded using a thermistor^40^.

Lake Müggelsee (Muggelsee), located in Berlin, Germany, was sampled between 8:00 and 9:00 at a depth of 0.5 m. The samples from 1985–1994 were collected manually using a mercury thermometer at a jetty 30 m from the shore, from 1994–2002 a YSI multiparameter sonde (6600 V2 sonde) was used at the same location, and from 2002–2009 the same sonde was deployed 300 m from the shore^41^.

Lake Constance is a large and deep lake bordering Germany, Switzerland and Austria. Water temperature was measured once or twice a month at the sub-surface (approximate depth: 0.01 m). Water temperatures were recorded in the morning usually between 9:00 and 10:00 using a thermometer from an open water site in the middle of Upper Lake Constance (water column depth at sampling site: 252 m) (ref. 42).

Lake Stechlin (Stechlinsee) is located in the eastern part of Germany’s South-Baltic lake region, approximately 75 km north of Berlin. Data on water temperature were provided by the German Meteorological Service (DWD, 1958–1996) and the Institute of Freshwater Ecology and Inland Fisheries (IGB, including predecessor institutions, 1970–2011), respectively. Vertical profiles were taken in the morning (10:00–12:00) at the deepest location of the lake (69 m). The DWD series consisted of monthly measurements in 2 m intervals from 0 to 20 m and then in 10 m intervals to the deepest point. Profiles in the IGB series were generally taken monthly, but were taken less often in the 1980 s and then were taken biweekly during the summer after 1991. In the epilimnion, profiles were taken at 2.5 m intervals (1.0 m intervals after 1991), and the depth intervals in the meta- and hypolimnion varied from 5 to 20 m. Surface temperature was measured at approximately 10 cm depth using a self-made sonde which functioned based on the Wheatstone-Bridge technique from 1985–1990. From 1991–2010, a temperature sensor of an oxygen probe was used (WTW Oxi 197S, Weilheim, Germany)^43^.

Lakes Vänern (Vanern), Mälaren (Malaren), Skärgölen (Skargolen), Stensjön (Stensjon), Remmarsjön (Remmarsjon), Allgjuttern, Brunnsjön (Brunnsjon), Övre Skärjön (Ovre Skarjon), Stora Envättern (Stora Envattern), Rotehogstjärnen (Rotehogstjarnen), Fiolen, St Skärsjön (St Skarsjon) and Fracksjön (Fracksjon), all located in Sweden were sampled monthly during the ice-free season, while Lakes Vättern (Vattern) and Erken, also in Sweden were sampled daily throughout the year. All lakes were sampled at a depth of 0.5 m, in addition to several other depths depending on the maximum lake depth. The samples were obtained from the central part of the lake above the deepest point except for the daily time series in Lake Vättern which were collected at a drinking water intake point in the eastern part of the lake. Water temperature sampling was performed using a thermometer in the Ruttner sampler for all lakes. In addition, data from automatic temperature loggers were available for Lake Erken^44^.

Lake Valkea-Kotinen, situated in southern Finland, was sampled weekly, and water temperatures were measured at the surface and depths of 1, 2, 3, 4, 5 and 6 m. Measurements were made in the deepest part of the lake (6.5 m) in the morning between 10:00 and 11:00 using YSI probes for oxygen and temperature^45^.

Lake Pääjärvi (Paajarvi), situated in southern Finland, was sampled monthly. Water temperature was measured at different depths from the top to the bottom of the lake. The measurements were made at the deepest point of the lake (85 m) before noon using YSI probes for temperature and oxygen.

Lakes Inari, Kallavesi, Kevojärvi (Kevojarvi) Lappajärvi (Lappjarvi), Päijänne (Paijanne), Pielavesi, Pielinen, and Saimaa are all located in Finland. Lakes Inari and Kevojärvi are located in the northernmost Lapland Province. Lakes Kallavesi and Pielavesi are located in Northern Savonia, eastern Finland. Lake Lappajärvi is located in the municipalities of Lappajärvi, Alajarvi, and Vimpeli, in southern Ostrobothnia, Finland. Lake Päijänne is located south of the city, Jyvaskyla, Lake Pielinen is located in North Karelia, and Lake Saimaa is located in southeastern Finland. All lakes were sampled daily at a depth of 0.2 m from sites close to the water level gauge. Water temperatures were collected at 8:00 with mercury thermometers (with an accuracy of 0.5 °C) prior to the 1990 s and with digital thermometers in the 1990 and 2000 s (ref. 46).

Lake Pyhäselkä (Pyhaselka), located in North Karelia, Finland, was sampled 0 to 3 times per month. The samples were obtained from four different locations on the same lake. These sites were Kaskesniemi, Noljakansaaret, Pyhäsaari, and Kokonluoto, with maximum depths of 38, 13, 53, and 65 m (ref. 47). Each site was sampled from the surface (1 m) to the bottom. Kaskesniemi was sampled at an interval of 2 to 4 m, Noljakansaaret at an interval of 2 m, and both Pyhäsaari and Kokonluoto at an interval of 5 m depth. Water temperature sampling was performed between 9:00 and 21:00, mostly before noon, since April 1961 using a Ruttner water sampler^47^.

Lake Võrtsjärv (Vortsjarv) in southern Estonia, was sampled twice daily at 8:00 and 20:00 until April 30 2011, and hourly since then, at the surface. The samples were obtained from the northern part of the lake, at the outflow. Water temperature sampling was performed manually with a mercury thermometer until April 30, 2011 (ref. 48).

Lake Peipsi (Peipus) on the border of Estonia and Russia, was sampled twice daily at 8:00 and 20:00, at a depth of 0.1 m (ref. 48). The surface water temperature was measured at Mustvee, Mehikoorma and Praaga hydrometric stations of Lake Peipsi. Water temperature sampling was conducted using a Celsius mercury thermometer. Since May 2009, an automatic station (VAISALA, MAWS110 and water temperature sensor QMT110) was used every hour for water temperature measurements.

### Middle East

Lake Kinneret in Israel was sampled weekly from 1969 and every 10 min from 1996 to present at a depth of 5 cm (surface). The samples were obtained from the central monitoring station of Lake Kinneret, approximately 42 m in depth. Water temperatures were recorded at 10:00 with a STD-12 (Applied Micro-systems), with an error of 0.005 degrees C. The equipment was replaced several times^49^.

### Asia

Lake Baikal, located in south of the Russian region of Siberia, was sampled approximately every 10–14 days. The lake was sampled at the following depths: 0, 10, 25, 50, 100, 250 m. The samples were obtained from a long-term station in the southern basin approximately 2.7 km offshore from Bol’shie Koty where the water depth is approximately 800 m (ref. 50). Water temperature sampling was performed usually in the morning and sometimes in the afternoon using a mercury thermometer in a VanDorn closing bottle^6^.

Lake Taihu, located near Shanghai, China was sampled monthly at a depth of 0.2–0.5 m. The samples were obtained at two locations near the lake centre and two locations in Meiliang Bay. Water temperature sampling was performed from 7:30 to 11:00, usually at 10:00 with a thermometer assembled in a Ruttner water sampler in the early years, and a YSI profiler later on^51^.

Lake Biwa is located in Shiga Prefecture northeast of Kyoto in Japan. Temperature data were collected near the deepest part of the lake using a Hydrolab Quanta thermistor deployed by the Lake Biwa Environmental Research Institute. Measurements were made twice a month at 0.5, 5, 10, 15, 20, 30, 40, 60, 80 m, and bottom (approximately 90–95 m) (ref. 52). The sampling method was fixed between 1985 and 2009, and some data are opened to the public through the official home page at http://www.pref.shiga.lg.jp/d/biwako-kankyo/lberi/.

### Africa

Lake Tanganyika is located at the border of four countries (Republic of Burundi, Democratic Republic of Congo, United Republic of Tanzania, Republic of Zambia). It was sampled weekly to twice a month from 0 to 100 m depth with an interval of 10 to 20 m. The samples were obtained from the pelagic area between 10:00 and 12:00. Water temperature sampling was performed using digital thermometers (YSI model 50B) by the Food and Agricultural Organization (FAO) of the United Nations from 1993–1996. From 2002–2005, sampling was done using a Seabird 19 CTD at Kigoma (Tanzania) by the Nyanza Project and the CLIMLAKE/CLIMFISH project^53^.

### Oceania

Twelve reservoirs, namely, Barossa, Beetaloo, Happy Valley, Hope Valley, Little Para, Millbrook, Mount Bold, Myponga, South Para, Tod, Kangaroo Creek Reservoir and Warren are all located in Adelaide, south Australia. All were sampled weekly except Mount Bold, South Para, and Tod which were sampled fortnightly. Hope Valley, Little Para, and Tod were sampled at a depth of 0 m, Warren at depths of 0, 5, and 10 m, Happy Valley at 0, 5, 10 and 15 m, Beetaloo at depths of 0, 10 and 15 m, Millbrook and South Para at 0, 10, and 20 m, Barossa at 0, 10, 15, 20 and 25 m, Mount Bold and Myponga at 0, 10, 20 and 30 m, and Kangaroo Creek Reservoir at 0, 10, and 30 m. All samples were obtained from sites adjacent to the dam wall. Water temperatures were recorded in the morning with a handheld YSI or Hydrolab thermistor^54^.

Maroonda, Silvan and Sugarloaf Reservoirs are located in Victoria, Australia. These reservoirs were sampled daily at a sampling depth of 0 m. Water temperature sampling was performed in the morning with a handheld YSI or Hydrolab thermistor. The samples were obtained from sites adjacent to the dam wall.

Cardinia, Greenvale, Upper Yarra and Yan Yean Reservoirs are located in Victoria, Australia. Data were collected at 2–5 min intervals at multiple depths from the surface to the bottom in increments from less than 1 m to approximately 5 m; surface water measurements were made at 0.5 m. Water temperatures were recorded using a Lake Diagnostic System (LDS) with PME thermistor chain typically moored at the deepest point in the reservoirs and close to the dam wall.

Burragorang, Cataract, Cordeaux, Fitzroy, and Wingecarribee are all reservoirs located in New South Wales, in southeast Australia. Burragorang and Wingecarribee were sampled weekly. Cataract was sampled fortnightly and, Cordeaux and Fitzroy were sampled monthly. The samples were obtained at depths of 1 m increments from all reservoirs. Samples were obtained from five sites in Burrangorang, specifically from 14 km south of the dam wall (0–79 m), 4 km from Butchers (0–33 m), 9 km south of the dam wall (0–51 m), at Woll arm (0–60 m), and 500 m south of the dam wall (0–100 m). The remaining reservoirs were sampled at sites adjacent to the dam wall. Water temperatures were recorded in the morning with a handheld YSI or Hydrolab thermistor. Although there were equipment upgrades over the years, there have been no substantive change to equipment^55^.

Lake Samsonvale (North Pine Dam) is located in Queensland, Australia. The samples were collected from the North Pine vertical profiler approximately 100 m upstream of North Pine Dam Wall and two thermistor strings (approximately 1.3 and 3.4 km upstream of the dam wall). These instruments are part of an autonomous monitoring program with the vertical profiler replacing the thermistor strings in May 2009. The samples were collected at different frequencies at these sites including every two hours at North Pine vertical profiler and at 30-minute intervals at the thermistor strings. The vertical profiler uses a YSI 6600v2 instrument while the thermistor string used Hydrolab instrumentation. Water temperatures were recorded at various depths at 1 m intervals from surface to bottom. Additionally, monthly grab samples were taken at 1 m intervals at the dam wall site using a variety of handheld instruments and more recently using a YSI 6600v2.

Water temperatures at a depth of 1 m were sampled from Lake Taupo every 2 to 3 weeks. Lake Taupo is centrally located on North Island, in the Taupo District, Waikato Region, New Zealand. Water temperatures were sampled from the deepest site in the centre at 11:00 with a conductivity-temperature-depth (CTD) profiler^56^.

Lake Rotorua, located in North Island, New Zealand, was sampled monthly at depths of 0–1 and 18 m. The samples were obtained from the southeast part of the lake. Water temperature sampling was performed in the daytime with a CTD profiler and a thermistor. From January 20 2005, sampling depth changed from 0 to 1 m (ref. 57).

Lake Tarawera, located in North Island, New Zealand, was sampled monthly from the subsurface (20 cm) to the bottom at 1 m intervals. The samples were obtained from a deep, mid-lake site. Water temperature sampling was performed at 11:00 with a Seabird CTD from 2003. Before 2003, a YSI 6000 probe was used^58^.

### Satellite surface water temperatures

We compiled satellite-derived surface water temperatures from 154 lakes (Figure 1). The methodology used for obtaining satellite-based lake surface temperatures largely follows the approach described in Schneider and Hook^11^. Only lakes that exhibited at least a 10×10 km area of pure water surface without any islands or shorelines were selected, in order to make sure that there was no contamination by land surfaces. We used data from two series of satellite instruments in order to maximize the sampling frequency and thus to provide more accurate estimates of the average lake surface temperature for each July, August, and September (JAS)/ January, February, and March (JFM) period. These were the Advanced Very High Resolution Radiometer (AVHRR) series and the Along Track Scanning Radiometer (ATSR-1, ATSR-2, Advanced ATSR) series.

The AVHRR series of satellite instruments has been measuring thermal infrared (TIR) radiation since the late 1970 s. For this study, we used data from the version 5 product of AVHRR Pathfinder collection^59^. This is a joint NASA/NOAA global reprocessing of the AVHRR data archive that provides twice daily temperatures of large inland water bodies worldwide at a spatial resolution of approximately 4 km. AVHRR Pathfinder data between 1985 and 2009 were used. We extracted a single pixel for every site and every day from the manually selected coordinates over each lake that maximized the distance from any shoreline and any islands. This avoided any possible bias due to contamination from mixed pixels containing both land and water. We only used night time data in this study. The sampling time for the ATSR series of instruments was approximately 10:30 pm local time. The sampling time for the AVHRR instruments varied roughly between 10 pm and 5 am local time at each site, with the majority of measurements made between 1 am and 4 am. Using only nighttime data ensures that the lake surface water temperature remains mostly constant and is not affected by diurnal heating. In addition, we performed cloud masking so that any pixels that were acquired at zenith angles of greater than 45 degrees were discarded. The AVHRR Pathfinder product has been rigorously validated in the past against *in situ* radiometer data over open oceans^60,61^ and against buoy observations over inland water bodies^11^.

Secondly, we used TIR data from the series of Along-Track Scanning Radiometers (ATSR). ATSR-1 began acquiring data in 1991, and was followed by ATSR-2 which was launched in 1995, and by the Advanced Along-Track Scanning Radiometer (AATSR) launched in 2002. ATSR-1, ATSR-2 and AATSR stopped acquiring useful data in 1996, 2003, and 2012, respectively. The ATSRs are multi-channel imaging radiometers with the primary purpose of providing highly accurate sea surface temperature data worldwide. The instruments provide a nominal pixel size of 1 km^2^ at the centre of nadir and about 1.5×2.0 km at the centre of the forward swath. At the coordinates selected for each site, we extracted a 3×3 pixel array of thermal infrared data for each nighttime overpass. We eliminated cloudy pixels from the time series of top-of-atmosphere brightness temperatures using a subset of the cloud tests provided with the ATSR product and complemented by a spatial homogeneity test that excluded any arrays where the standard deviation of the 3×3 pixel array was greater than 0.5 °C.

We used the satellite data to calculate bulk water temperatures from skin temperatures using sensor-specific correction factors derived from the Laurentian Great Lakes^11^. We computed water skin temperature from the brightness temperatures using the dual-view operational split-window coefficients provided for ATSR^62^. ATSR data has also been extensively validated over the oceans and over the Lake Tahoe (CA/NV) validation site operated by the Jet Propulsion Laboratory^63^. We compiled time series from both series of satellite instruments independently at all 141 sites, with AVHRR data available for the period 1985–2009 and ATSR data available for the period 1991–2009. We then merged the cloud-free observations from both series of instruments into a single dataset to maximize the sampling frequency. This allows for more accurate estimates of the 3-month summer means.

### Geomorphometric characteristics

We collected latitude, longitude, elevation, surface area, volume, mean depth, and maximum depth for each lake, if available (Table 1). Although these values are presented as static, we note that these variables could have varied during the 1985–2009 period, especially for reservoir systems. Whenever possible, metadata for each lake were obtained from the original data provider. For many of the lakes sampled using satellite imagery, very little morphometric information was readily available. In these cases, we conducted searches in the primary literature, online web sources (ILEC (http://www.ilec.or.jp/en/), IAGLR (http://www.iaglr.org/), LakeNet (http://www.worldlakes.org/), Wikipedia (http://en.wikipedia.org/wiki/Main_Page)), Google Earth, or contacted colleagues in the relevant countries to obtain information.

### Air temperatures

We obtained mean, minimum, maximum, and diurnal temperature range (defined as the difference between daytime maximum and nighttime minimum temperatures) for summer, winter, and annual air temperature data for each lake from two separate sources: the Climatic Research Unit (CRU) at the University of East Anglia, United Kingdom and the National Centers for Environmental Prediction (NCEP). We obtained the CRU data from version 3.21 of their dataset using 0.5-degree resolution grid cells^64^. These data are monthly gridded files calculated from observations. Lake values were the mean of all months within the appropriate time period to match the sampling period (e.g., July-August-September for northern hemisphere summer, January-February-March for northern hemisphere winter), see Section G below) for a given year for the single grid cell that contained the sampling location for the lake. Exceptions included large bodies of water, such as Lake Superior and the Caspian Sea, which included an average of the grid cells that surrounded these lakes (because we used a land-based air temperature dataset, grid cells that were over only water did not have data).

The air temperature data we obtained from NCEP represent version 2 of the NCEP reanalysis data^65^. We retrieved air temperature data at a height of 2 m for each lake using the RNCEP package, a user-friendly package for use in the R programming language^66^. Air temperatures for the NCEP reanalysis data are projected onto a global T62 Gaussian grid of 192 equally spaced points in longitude and 94 unequally spaced points in latitude^65^. For lakes smaller than one cell on the T62 grid, we extracted data for the cell containing the latitude and longitude representing the approximate center of the lake. For lakes larger than one cell size on the grid, we aggregated all cells within a geometric square representing the surface area of the lake. Finally, we aggregated these data into summer means using the RNCEP.aggregate function^66^.

### Solar, longwave and total radiation

Summer and winter surface solar (i.e., shortwave) and longwave radiation data are a satellite product taken from the NASA/Global Energy and Water Cycle Experiment (GEWEX) Surface Radiation Budget (SRB) radiation dataset, version 3.0 (obtained from the NASA Langley Research Center Atmospheric Science Data Center). The data are generated on the basis of the International Satellite Cloud Climatology Project (ISCCP) DX radiance and cloud parameters^67^, using an updated version of the University of Maryland’s shortwave flux algorithm and an adaptation of the longwave algorithm. The dataset has a spatial resolution of 1°× 1° and temporal resolution of 3 h. In addition to the SRB incoming shortwave radiation (*SW*) and longwave radiation (*LW*), we also provide estimates of the total incoming radiation (*R*_*tot*_=0.93*SW+0.97*LW) that is absorbed by a dark, water surface under ice-free conditions (i.e., an assumed shortwave albedo of 7% and longwave emissivity of 0.97).

The SRB radiation data have been shown to be one of the best global radiation datasets and exhibit smaller biases than reanalysis data and other satellite products. For example, Gui *et al.* ^68^ assessed SRB solar radiation data using ground measurements collected at 36 globally distributed sites from 2000–2002. Results indicate that the downwelling solar irradiance shows good overall agreement with ground measurements, except for a few large biases in Southeast Asia (Sukothai, Bukit, and Kogma). These biases range from −90.2 W m^−2^ (−22%) at Sukothai to +45.8 W m^−2^ (+12.8%) at Bukit when comparing SRB solar radiation data with ground observations. Overall, however, the SRB data show a lower mean bias across 36 global sites (−5.5 W m^−2^; −1.9%) (ref. 68). Markovic *et al.* ^69^ also evaluated the SRB dataset at six surface radiation sites from the United States surface radiation (SURFRAD) network and found good agreement with the SRB data across the annual cycle.

### Cloud cover

Instrumentation measuring global cloud cover was rarely continuous during the full observational period of this study (1985–2009). The NOAA 5-channel Advanced Very High Resolution Radiometer (AVHRR) cloud imagery record began in 1981, and cloud statistics derived using Pathfinder Atmosphere’s Extended (PATMOS-x) processing system are available with global coverage^70^. To calculate seasonal means for this study we used temporally interpolated values representing 13:30 local time for *cloud amount* (fractional cloud cover) from a 1° re-gridded version of this dataset^71^. We calculated mean cloud cover (0–1) during the summer and winter periods using the average of these daytime values for 1985–2009.

### Summer mean surface water temperature calculations

We calculated summer mean temperatures for a 3-month period. Generally, for lakes situated in the Northern Hemisphere we used the period of 1 July—30 September (JAS); whereas, in the Southern Hemisphere, we used 1 January—31 March (JFM). Exceptions were latitudes less than 23.5°, for which the JAS metric was used south of the equator and the JFM metric was used north of the equator. This was done in order to avoid the cloudy wet season in the tropics and instead collect data during the dry season, which allows for an increased number of cloud-free satellite observations^11^. We use the term ‘summer’ throughout to refer to these time periods (JAS or JFM) depending upon location of the lake. The only exception to this time period was the *in situ* data for Toolik Lake, Alaska, for which we used June-August because of the lack of data available for September due to the early onset of winter at this high latitude. The term ‘winter’ refers to the 3-month period opposite ‘summer’ in the calendar year.

#### *In situ* mean calculations

Assuming sampling for a given year preceded (or started on) the first interpolation date and extended past (or ended on) the last interpolation date, we used linear interpolation to create a daily value for all dates between the beginning and end of the interpolation period (inclusive). The summer mean was calculated by finding the mean of all of these daily values beginning on the first day of July and ending on the last day of September (for Northern Hemisphere lakes). We also calculated gap lengths, which were the intervals in days between sequential sampling times. Gaps were calculated only for the sampling points that existed within the seasonal bounds. We defined the maximum gap as the maximum of all gaps for a single year. The mean gap was the mean of all gaps for a single year. If sampling did not extend to or beyond the start and end dates of the interpolation period, we used a curve-fitting extrapolation routine (see below).

#### Extrapolation of observations for shortened sampling periods

Where necessary, we used a curve-fitting extrapolation technique to extend daily *in situ* observations to the bounds of the summer mean period. More specifically, we used temperature estimates from lake-specific curves to populate daily values for the missing part of the interpolation period. To generate the curve, we pooled all temperature values (for a given depth and given lake) according to day-of-year (DoY), and fit a curve of the type ‘temperature=a*DoY^2+b*DoY+c’. This equation yielded a generic representation of the seasonal pattern in the data. For years that did not have data that covered the full interpolation period, we used the curve fit to extrapolate values preceding the first sample point (if applicable) or beyond the last sample point (if applicable), or in some cases, both. The curve was adjusted along the Y (temperature) axis by modifying the value of c in the equation to intersect the first sample point (if missing data appeared during the early portion of the interpolation period) or last sample point (if there were missing data in the latter part of the interpolation period), and daily values were taken from the equation for the portion(s) missing from the record. When this routine was used, we made a notation in the log file describing if extrapolation was used for the beginning and/or end period and we included the equation of the curve (See Data Records).

#### Satellite mean calculations

As the satellite observations generally are not distributed evenly throughout each 3-month period, we used a robust locally weighted regression smoothing (LOWESS) approach^72^ on a year-by-year basis for each time series. This technique provides a continuous temperature estimate and is robust against outliers in the observations helping to avoid sampling bias in the calculation of the 3-month mean. We computed a separate LOWESS fit for the temperature curve of each calendar year and at each inland water body. We subsequently computed the mean temperature of a 3-month period over all 92 daily values obtained from the fitted LOWESS curve. An average temperature was only computed if at least 20 valid cloud-free satellite retrievals were available in a given 3-month period, to ensure that the observations adequately characterize the temperature curve.

## Data Records

The data are available in two comma delimited text files (Data Citation 1). The first, GLTClakeinformation.csv, contains information for each lake, who collected the data, lake location and surroundings. The second table (Table 2), GLTCvalues.csv, contains the actual values and may be linked to the lake information via the siteID column. A zip folder of processing log files is included, called GLTC_means_processing_log_files.zip. This folder contains text files that explain any extrapolation performed as part of the summer mean calculations, including the parabolic equation used for the extrapolation, the time period covered, and the R^2^ fit to the data. Data used in this study have not previously been published, however, methods of collection and manipulation for harmonization are described in detail for each lake above and in the metadata. Both data entities are documented in the Ecological Metadata Language (EML) and are archived in the Long Term Ecological Research Network Information System and in the R package ‘laketemps’. Both data and metadata can be accessed at http://portal.lternet.edu/, https://www.dataone.org/ and in the R package ‘laketemps’.

## Technical Validation

### Quality control and assurance of *in situ* data

In order to efficiently assemble a complete product from the disparate data sources used for this dataset, we composed a set of scripts in the R programming language^73^. After the assembly of the dataset was complete, we examined the distribution of mean lake temperatures using a series of histograms and maps. The histograms allowed us to identify outliers while the maps provided geographic plots that could be examined for unusual observations or spatial patterns that might indicate technical errors during the assembly of the dataset. To maximize our chances of identifying erroneous values, the histograms and maps were examined by all co-authors. To ensure that the geomorphometric data in the completed dataset were accurate we had several individuals independently check the values against reputable sources.

The final step taken to assure the quality of our assembled dataset was to share the derived mean summer temperatures and geomorphometric data with the original data providers. We sent an email to each of the data providers containing the relevant data for their lake(s) along with a request to validate the values that we have included in the completed dataset. This final step was probably the most important as it allowed us to get feedback from the individuals that are most familiar with the lakes in our dataset. Whenever there were inconsistencies in data, we worked directly with the original data provider to resolve the issue and update the data accordingly.

### Assessment of sampling gap impacts on temperature trends

**Figure 3:**
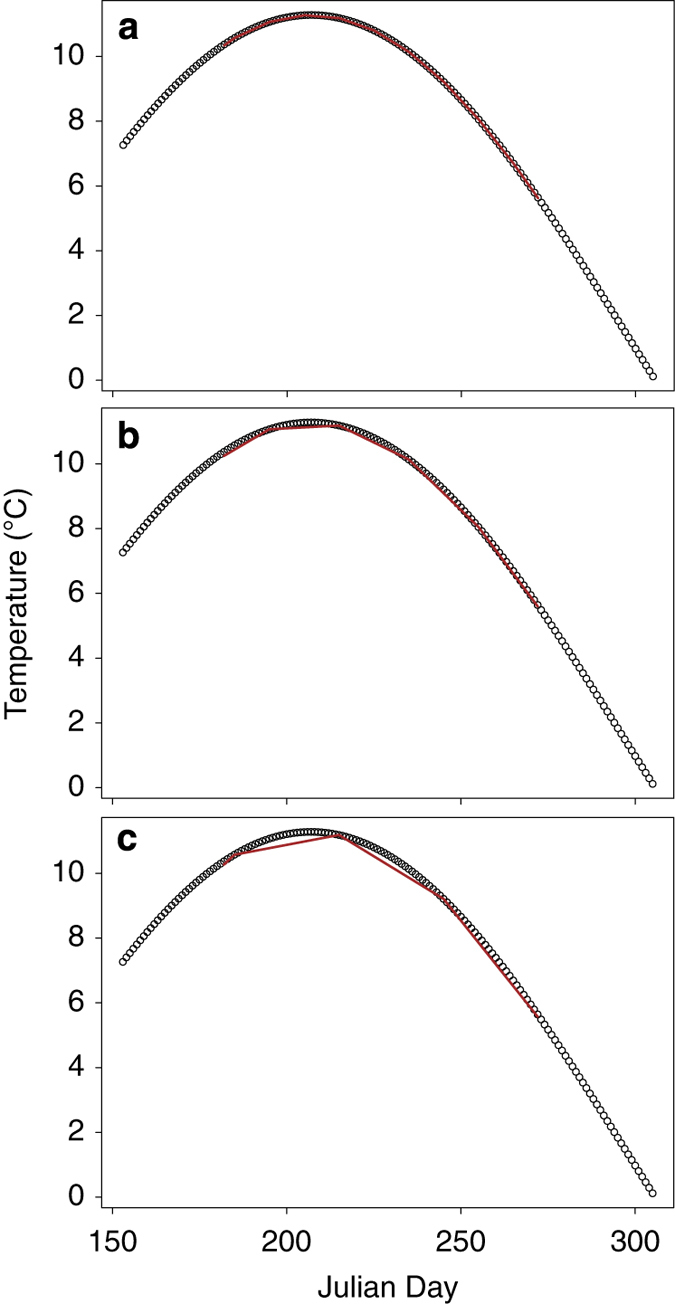
Linear interpolation was used to calculate daily temperature estimates for July, August, and September for each *in situ* sampled lake. The mean summer temperature for each lake was then calculated by taking the mean of those daily estimates. The figure shows interpolation conducted with temperature measurements conducted at an interval of once every 10 days (panel **a**), once every 20 days (panel **b**), or once every 30 days (panel **c**). At high sampling intervals (panel **c**), linear interpolation can underestimate daily temperatures, leading to an underestimate of the mean summer temperature.

**Figure 4:**
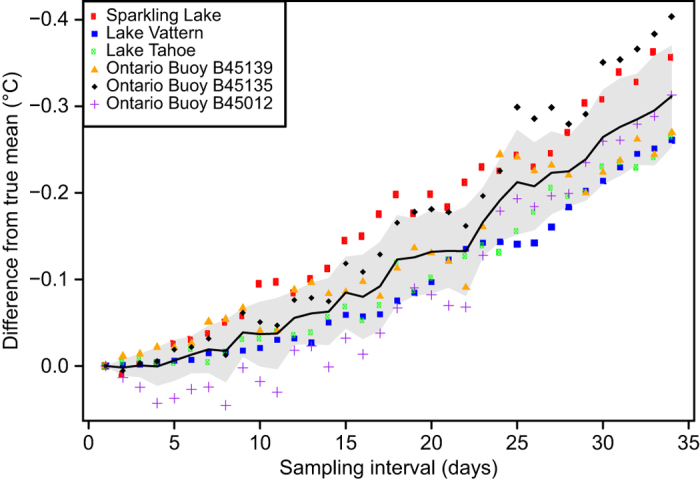
The relationship between sampling interval and error in our mean summer temperature estimates for *in situ* sampled lakes. A sampling interval of 1 indicates that temperature measurements were collected daily, while an interval of 34 indicates one temperature measurement every 34 days. Temperature measurements collected with a sampling interval of 1 were considered to have no error and served as a basis for comparison with data collected at higher sampling intervals.

**Figure 5:**
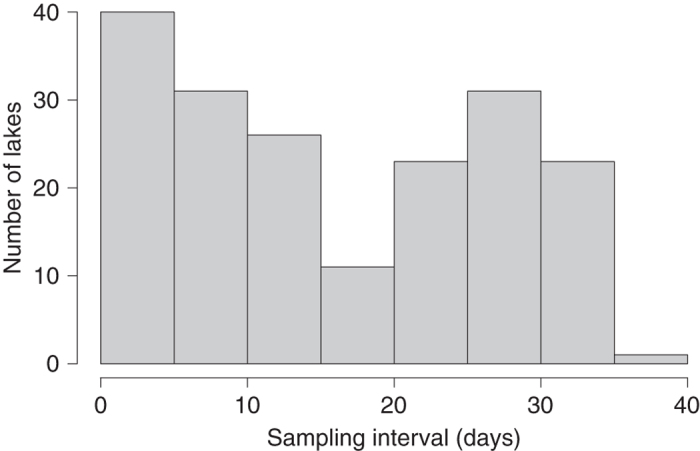
A histogram of the mean sampling intervals for the *in situ* sampled lakes found in our dataset. A sampling interval of 1 indicates that temperature measurements were collected daily, while an interval of 40 indicates one temperature measurement every 40 days.

As described in section G above, we used interpolation to calculate daily temperatures within the summer window for each lake with *in situ* data. The mean summer surface temperature for each lake was then calculated by taking the mean of those daily estimates. While this approach allowed us to standardize the methodology used for calculating mean summer temperatures across our lakes, we were concerned that mean temperatures calculated for lakes with longer sampling intervals (e.g., one temperature measurement each week) would have more error than lakes with shorter sampling intervals (e.g., daily temperature measurements). Our rationale for this concern was that linear interpolation can produce an underestimate of mean summer surface temperatures when few data points are available, and the temperature trajectory is parabolic (Figure 3). In order to estimate the amount of error attributable to differences in sampling interval, we designed bootstrap simulations in the R programming language. For these simulations, we used temperature records from six sites on four lakes that had daily temperature data available (Figure 3). For each site, we constructed simulated temperature datasets consisting of a subset of the daily temperature observations. We used sampling intervals ranging from once per day to once every 34 days and constructed 20000 simulated datasets for each sampling interval. The date of the first sample for each of the 20000 datasets was chosen as a random date in June and then the following samples were equally spaced in time starting from that date. Therefore, variation among the simulated datasets for each sampling interval was driven by random variation in the choice of the initial sampling date. We then used linear interpolation to calculate daily temperatures for July, August, and September. The mean of these daily temperatures represented the annual mean summer temperature. We treated the mean annual summer temperature calculated with a sampling interval of 1 (daily measurements) as the ‘true’ mean temperature. For all of the other sampling intervals we calculated the difference between the true mean and the mean calculated with interpolation. The difference was calculated by year for each simulated dataset. We then took the average error for the 20,000 simulated datasets for each sampling interval and plotted the results. In this manner, we were able to construct a relationship between sampling interval and error in our temperature estimates (Figure 4). Our results demonstrated that although errors in our temperature estimates did increase at large sampling intervals, they were relatively modest, even at a sampling interval of once every 34 days (−0.25 to −0.4 °C; Figure 3). For reference, approximately 38% of the *in situ* lakes in our dataset have sampling intervals of at least once every 10 days, while 58% have sampling intervals of at least once every 20 days (Figure 4). Only 13% have sampling intervals≥once every 30 days (Figure 5).

### Validation of satellite data with *in situ* measurements

We validated the satellite data with the *in situ* data by conducting four analyses. First, we compared summertime mean values for the 11 lakes that had both satellite and *in situ* data (Biwa, Baikal, Erie, Garda, Geneva, Huron, Michigan, Peipsi, Superior, Tahoe, Taihu). Second, we compared interannual anomalies. The RMSE between satellite and *in situ* measurements for the 11 long-term summer mean lake surface temperatures was 1.15 °C. Differences between the two mean values are assumed to be the result of different sampling times (often day versus night), different sampling frequencies, measurement errors, and differences between skin and bulk water temperatures. When we compared interannual anomalies (average deviations from the 1985–2009 mean), the RMSE declined to 0.72 °C. Third, we compared trends from the *in situ* data for 1985–2009. Trends that we calculated from temporally-matched satellite and *in situ* summer means for the 11 lakes had an RMSE difference of 0.03 °C per year.

Fourth, on a small subset of the data, Schneider and Hook^11^ used high-frequency buoy measurements to conduct a more direct comparison of water temperature trends that eliminated differences in timing and observation frequency. Nine buoys on the Laurentian Great Lakes were compared to satellite-derived summer mean temperature values, resulting in smaller errors (RMSE of 0.43 °C) and negligible biases for satellite estimates. Schneider and Hook^11^ also applied two additional methods to summer means from these nine sites to confirm that these biases did not negatively affect the computed trends: Firstly a direct validation of satellite-derived trends against corresponding trends obtained for *in situ* observations at buoys was carried out and the results indicated a good correspondence with an RMSE of 0.013 °C per year. Secondly, a linear regression analysis of time series of annual biases revealed no significant trend, indicating that satellite calibration drift did not adversely affect trends obtained from the data.

## Additional information

**How to cite this article:** Sharma, S. *et al.* A global database of lake surface temperatures collected by *in situ* and satellite methods from 1985–2009. *Sci. Data* 2:150008 doi: 10.1038/sdata.2015.8 (2015).

## Supplementary Material



## Figures and Tables

**Table 1:** Data labels and description for metadata file (lakeinformation.csv).

**Data label**	**Description**	**Proportion of lakes for which data were available or estimated based on calculations or maps**
Lake_name	Lake name as provided by the contributor. In the case where a lake name appears multiple times (followed by a period and additional text), this represents multiple sites within that lake.	100%
Other_names	Some lakes may have additional names, based on borders shared between countries, differences in spellings, or alternative names found when locating the lakes on Google Earth.	(49 lakes) 17%
lake_or_reservoir	Whether a water body is classified as a lake or as reservoir/dam.	100%
location	Country or countries along the shoreline.	100%
region	Geographic region of the world.	100%
latitude	Latitude coordinates in decimal degrees. For *in situ*-sampled lakes, these were frequently derived from Google Earth or from a shoreline location.	100%
longitude	Longitude coordinates in decimal degrees. For *in situ*-sampled lakes, these were frequently derived from Google Earth or from a shoreline location.	100%
geospatial accuracy_km	Estimate of the accuracy of the latitude and longitude point. For satellite-sampled lakes, accuracy of the georeferencing is roughly 0.5 km for the ATSR series of instruments and about 2 km for the AVHRR series; because these series are combined, 2 km is listed in the database. Only lakes that exhibited a 10×10 km area of pure water surface were used to ensure that inaccuracies in georeferencing would not introduce any contamination by land surfaces. For *in situ*-sampled lakes, in most cases the coordinates reflect the general lake or a shoreline location and may not necessarily reflect the exact water temperature sampling location.	100%
elevation	Elevation of the water level.	100%
mean_depth_m	Mean depth of the lake.	93%
max_depth_m	Maximum depth of the lake.	95%
surface_area_km2	Surface area of the lake.	100%
volume_km3	Volume of the lake.	93%
source	Whether surface water temperature is derived from *in situ* or satellite measurements	100%
sampling_depth	The water depth at which temperature measurements were taken. Skin-derived bulk temperature is approximately equivalent to 1 m depth.	100%
sampling_time_of_day	Time of the day during which temperature measurements were taken. In general, *in situ* sampling was done during the day (8:00–16:00) while satellite measurements were most commonly done between 1:00 and 4:00. Measurements were made with automated systems, such as buoys, are listed as ‘continuous’.	100%
time_period	The 3-month period during which data was averaged to create a summer surface water temperature, using abbreviations from the first letter of each month’s name. This period also coincides with summer data for the meteorological variables associated with the lake.	100%
contributor	Names and contact information for people who contributed the data.	100%

**Table 2:** Data labels and description for data values file (values.csv).

**Value label—change to this**	**Value description**
Lake_Temp_Summer_InSitu	Mean lake surface water temperatures for the summer 3-month period collected by *in situ* methods in degree centigrade
Air_Temp_Mean_Summer_NCEP	Mean air temperature for the summer 3-month period in degree centigrade, from NCEP
Air_Temp_Mean_Annual_NCEP	Annual mean air temperature in degree centigrade, from NCEP
Air_Temp_Mean_Winter_NCEP	Mean air temperature for the winter 3-month period in degree centigrade, from NCEP
Lake_Temp_Summer_Satellite	Mean lake surface water temperatures for the summer 3-month period collected by satellite methods in degree centigrade
Radiation_Shortwave_Summer	Amount of incoming shortwave radiation, during the summer 3-month period in watts per square meter, from SRB
Radiation_Shortwave_Winter	Amount of incoming shortwave radiation, during the winter 3-month period in watts per square meter, from SRB
Radiation_Longwave_Summer	Amount of incoming longwave radiation, during the summer 3-month period in watts per square meter, from SRB
Radiation_Longwave_Winter	Amount of incoming longwave radiation, during the winter 3-month period in watts per square meter, from SRB
Radiation_Total_Summer	Total amount of incoming radiation, as calculated from shortwave and longwave in watts per square meter, during the summer 3-month period
Radiation_Total_Winter	Total amount of incoming radiation, as calculated from shortwave and longwave in watts per square meter, during the winter 3-month period
Radiation_Shortwave_Annual	Amount of incoming shortwave radiation during the year in watts per square meter, from SRB
Radiation_Longwave_Annual	Amount of incoming longwave radiation during the year in watts per square meter, from SRB
Radiation_Total_Annual	Total amount of incoming radiation during the year, as calculated from annual shortwave and longwave in watts per square meter
Cloud_Cover_Summer	Mean % cloud cover for the summer 3-month period, from PATMOS
Cloud_Cover_Winter	Mean % cloud cover for the winter 3-month period, from PATMOS
Cloud_Cover_Annual	Annual mean of % cloud cover, from PATMOS
Air_Temp_Max_Summer_CRU	Mean of the daily maximum air temperatures for the summer 3-month period in degree centigrade, from CRU
Air_Temp_Max_Annual_CRU	Mean of the daily maximum air temperatures for the year in degree centigrade, from CRU
Air_Temp_Max_Winter_CRU	Mean of the daily maximum air temperatures for the winter 3-month period in degree centigrade, from CRU
Air_Temp_Mean_Summer_CRU	Mean air temperature for the summer 3-month period in degree centigrade, from CRU
Air_Temp_Mean_Annual_CRU	Annual mean air temperature in degree centigrade, from CRU
Air_Temp_Mean_Winter_CRU	Mean air temperature for the winter 3-month period in degree centigrade, from CRU
Air_Temp_Min_Summer_CRU	Mean of the daily minimum air temperatures for the summer 3-month period in degree centigrade, from CRU
Air_Temp_Min_Annual_CRU	Annual mean of the daily minimum air temperatures in degree centigrade, from CRU
Air_Temp_Min_Winter_CRU	Mean of the daily minimum air temperatures for the winter 3-month period in degree centigrade, from CRU
Air_Temp_DTR_Summer_CRU	Mean of the daily diurnal temperature range (DTR) (calculated as the daily maximum minus the daily minimum) for the summer 3-month period in degree centigrade
Air_Temp_DTR_Annual_CRU	Annual mean of the daily diurnal temperature range (DTR) (calculated as the daily maximum minus the daily minimum) in degree centigrade
Air_Temp_DTR_Winter_CRU	Mean of the daily diurnal temperature range (DTR) (calculated as the daily maximum minus the daily minimum) for the winter 3-month period in degree centigrade
See the text for how ‘summer’ is defined for each lake location. ‘Winter’ is defined as the 3-month period opposite to ‘summer’ in the calendar year (e.g., winter is January-February-March for lakes where summer is July-August-September, and vice versa). Air temperatures were obtained from National Centers for Environmental Prediction (NCEP) and Climatic Research Unit (CRU), radiation from Surface Radiation Budget (SRB) and cloud cover from Advanced Very High Resolution Radiometer Pathfinder Atmosphere Extended dataset (PATMOS).	
